# Protective Predictors Associated With Posttraumatic Stress and Psychological Distress in Chinese Nurses During the Outbreak of COVID-19

**DOI:** 10.3389/fpsyg.2021.684222

**Published:** 2021-05-26

**Authors:** Lu Xia, Yajun Yan, Daxing Wu

**Affiliations:** ^1^Medical Psychological Center, The Second Xiangya Hospital, Central South University, Changsha, China; ^2^Medical Psychological Institute of Central South University, Changsha, China; ^3^National Clinical Research Center on Mental Disorders, Changsha, China

**Keywords:** COVID-19, posttraumatic stress, psychological distress, prevalence, protective predictor

## Abstract

**Background:** The novel coronavirus disease 2019 (COVID-19) posed an unprecedented threat to Chinese healthcare professionals. Nevertheless, few studies notably focused on the mental health conditions of nurses and explored protective factors to prevent posttraumatic stress and psychological distress. This study aimed to explore the prevalence and the predictive factors especially defensive predictors associated with posttraumatic stress and psychological distress in nurses during the COVID-19 pandemic.

**Methods:** In this online study, 1,728 nurses (~77.5% came from the COVID-19 pandemic frontline) were included in the final analysis. Posttraumatic stress disorder checklist for Diagnostic and Statistical Manual of Mental Disorders, 5th Edition (PCL-5) and Self-Reporting Questionnaire (SRQ) was used to assess posttraumatic stress and psychological distress.

**Results:** The results demonstrated that the prevalence of posttraumatic stress and psychological distress in nurses throughout China between February 1, 2020 and February 13, 2020 was 39.12 and 24.36%, respectively. Multivariate logistic regression indicated that insomnia, high panic intensity, and high impact of the COVID-19 pandemic were risk predictors of posttraumatic stress and psychological distress in nurses. Married participants had a 1.58 times increased risk of having posttraumatic stress when compared with the single participants. Frontline medical staff were more likely to suffer from psychological distress. The adequate exercise was a protective predictor of psychological distress [adjusted odds ratio (AOR) = 0.655, 95% CI = 0.486–0.883], but not with posttraumatic stress. High-quality diet was a protective predictor of posttraumatic stress (AOR = 0.112, 95% CI = 0.037–0.336) and psychological distress (AOR = 0.083, 95% CI = 0.028–0.247).

**Conclusions:** Our study revealed the prevalence and factors associated with posttraumatic stress and psychological distress in nurses during the COVID-19 pandemic. Low panic intensity, low level of impact, satisfactory sleep, adequate exercise, and better diet were protective factors of posttraumatic stress and psychological distress. It indicated that the psychological status of nurses (particularly those from the COVID-19 pandemic frontline) should be monitored, and protective factors associated with posttraumatic stress and psychological distress should be increased.

## Introduction

In December 2019, the novel coronavirus disease 2019 (COVID-19) outbreak which spread globally and resulted in a worldwide pandemic emerged in Wuhan, Hubei province, China (Li Q. et al., 2020). It had never been found before in humans or animals and had subsequently garnered attention around the world following the rapid increase of new cases (Wang et al., 2020). The virus belongs to the coronavirus family, which could cause respiratory infections in humans that resembled the common cold, as well as a lethal illness similar to that associated with the Middle East respiratory syndrome and severe acute respiratory syndrome (Carver and Phillips, 2020). Because of its high infectivity and uncertainty, as well as its high mortality rate, no adequate treatment was available in the short term.

During the 2nd week in March 2021, new cases continued to rise globally, increasing by 10% to over 3 million new reported cases. The Americas and Europe continued to account for over 80% of new cases and new deaths (World Health Organization, 2021). According to data released by the National Health Commission of China, the number of confirmed cases in mainland China had decreased to 164 as of March 18, 2021, but overseas imported cases had been increasing (The National Health Commission of China, 2021). As the source region of the COVID-19 pandemic, Chinese medical work still faced heavy burdens and great challenges.

This pandemic posed a huge challenge to healthcare workers (HCWs) because of successive waves of infections with short recovery phases. The COVID-19 outbreak brought a negative psychological impact on the medical staff, such as stress, depression, anxiety, and worse sleep quality (Huang and Zhao, 2020; Zhu et al., 2020). Nurses were associated with a high incidence of secondary traumatic stress even in medical routine work (Beck, 2011; Duffy et al., 2015). A recent study showed that nurses had a higher level of burnout, insomnia, and anxiety in comparison with physicians. The fear of infecting others and the fear of being infected were the only direct factors related to the COVID-19 and associated with the positive variation in nurses' symptoms of depression, anxiety, and stress (Sampaio et al., 2021). Frontline medical workers of preventing the COVID-19 had been facing more enormous pressure, including a high risk of infection and inadequate protection from contamination, overwork, frustration, discrimination, isolation, patients with negative emotions, a lack of contact with their families, and exhaustion (Kang et al., 2020).

Posttraumatic stress disorder (PTSD) is a mental health condition that could follow exposure to stressful life events. Per the Diagnostic and Statistical Manual of Mental Disorders, 5th Edition (DSM-5), symptoms of PTSD included intrusive recollections of the adverse event, avoidance behavior, a sense of ongoing threat and hypervigilance, and negative alterations in cognition and mood (American Psychiatric Association, 2013). Efficacious treatments for PTSD exist (Foa et al., 2008). Understanding on risk factors that temporally preceded posttraumatic stress (PTS) symptomatology is crucially vital to develop preventative interventions; this is important in providing effective interventions for PTSD prevention (Qi et al., 2016). Psychological distress (PD) is a heterogeneous range of symptoms, which include anxiety, anguish, depression, and demoralization (Massé, 2000; Ridner, 2004). It might meet the diagnostic criteria for major depression or an anxiety disorder when such symptoms are severe.

Previous studies provided evidence that frontline HCWs experienced PD and PTS during the severe acute respiratory syndrome (SARS) outbreak (Tam et al., 2004; Wu et al., 2009). Chen revealed that gender, education level, salary, work stress, job risk, depression, anxiety, insomnia, and PTS syndrome during the epidemic period were predictors of PTS and PD (Chen et al., 2020). A meta-analysis also showed that PTSD was associated with diet, exercise, and healthier habits including sleeping (van den Berk-Clark et al., 2018).

Based on the above research evidence, we assumed that the mental health of nurses might also be egregiously affected and predicted that the prevalence of PTS and PD in nurses was high during the COVID-19 pandemic, also that diet, exercise, and sleep condition were predictors associated with PTS and PD. We evaluated the prevalence of PTS and PD in nurses during the COVID-19 pandemic and mental health among nurses by quantifying the symptoms of insomnia, panic intensity, and other aspects and by analyzing influencing factors of these symptoms. The researchers hoped that the results of this study could provide support for the targeted interventions of the mental health of nurses during the outbreak.

## Materials and Methods

### Procedure and Participants

To prevent the spread of COVID-19 through contact, we used a survey based on the large Internet marketing research company in China (https://www.wjx.cn/) following the research methodology guideline (Andrews et al., 2003) to collect data. This web-based survey of COVID-19 was conducted on the Internet through the WeChat public platform. All participants using WeChat could see this survey and answered the questionnaire by scanning the two-dimensional barcodes of the questionnaire address or clicking the relevant link. The (deleted for blind review) institutional review board approved the ethical and scientific validity of this study. Electronic informed consent was obtained from each participant before starting the investigation. This web-based questionnaire was completely voluntary and non-commercial. Participants could withdraw from the survey at any moment without providing any justification.

From February 1 to February 13, 2020, 1,970 online questionnaires were collected from nurses nationwide. A total of 1,728 nurses were included in the final analysis after excluding the 242 questionnaires with wrong information (87.71% response rate). Approximately 77.5% of the samples were frontline nurses in the COVID-19 pandemic.

### Measures

#### Sociodemographic Variables

The questionnaire set included a brief survey to collect sociodemographic and context characteristics with the work of preventing COVID-19. According to the Italy model (Carlucci et al., 2020), sociodemographic variables included age, gender (male or female), marital status (single, married, divorced, or widowed), and the role in pandemic prevention. The role in pandemic prevention included the following three types: (1) Frontline (those who directly provided services to confirmed or suspected patients with COVID-19); (2) Medical Reserve Corps (those who probably contacted confirmed or suspected patients with COVID-19); and (3) Medical Routine Work (those who were less likely directly servicing confirmed or suspected patients with COVID-19).

We provided four items to assess the subjective influence of the COVID-19 pandemic: (1) How long are you exposed to the COVID-19 pandemic (the time in contact with the outbreak scene): always staying in the epidemic scene, most of the time at the scene of the epidemic, a small part of the time at the scene of the epidemic, not at the epidemic scene; (2) How long do you spend browsing COVID-19-related information per day: 0–2 h, 3–5 h, 6–10 h, 11–15 h, 16–24 h; (3) Do you experience panic during the COVID-19 pandemic: never, occasionally, sometimes, often, always; (4) To what extent has the current outbreak affected you: no impact, mild impact, moderate impact, severe impact, and extreme impact.

We used three items to evaluate the self-report physical conditions: (1) sleep: Insomnia was a common disorder after stress and was evaluated by the Insomnia Severity Index (ISI); (2) exercise: Exercise habits are defined as meeting the WHO physical activity recommendations for adults aged 18–64 years old; (3) diet: Diet was measured according to self-reports using the healthy eating index.

#### Self-Reporting Questionnaire

The SRQ was designed by the WHO as a cost-effective screening instrument for common mental disorders (Beusenberg and Orley, 1994). It consisted of 20 short questions that required a “yes” or “no” response, depending on the presence or absence of symptoms in the past month. The Chinese version of SRQ-20 comprised of three subscales: depressive symptoms (10 items), anxiety and somatic symptoms (five items), and somatic and anxiety symptoms (five items). It exhibited satisfactory psychometric properties as a screening tool for PD (Chen et al., 2009). A cutoff of seven was recommended according to WHO for evaluation of PD (Beusenberg and Orley, 1994). The measurement model of the SRQ-20 was evaluated using confirmatory factor analysis (CFA). The criteria for assessing adequate model-fit included: the normed fit index (NFI) = 0.857, the comparative fit index (CFI) = 0.873, the incremental fit index (IFI) = 0.873, and the relative fit index (RFI) = 0.837. The SRQ-20 model was acceptable. SRQ-20 had good internal consistency with Cronbach's α coefficients of more than 0.87 in our sample.

#### Posttraumatic Stress Disorder Checklist for DSM-5

The PTSD checklist for DSM-5 (PCL-5) was a 20-item self-report measure designed to mirror each DSM-5 PTSD symptom (Blevins et al., 2015). A total-symptom score of 0–80 could be obtained by summing up the items. The PCL-5 comprised of four subscales: intrusion symptoms (five items), avoidance symptoms (two items), cognition and mood symptoms (seven items), and arousal and reactivity symptoms (six items). It scored on a five-point Likert scale from 0 (*not at all*) to 4 (e*xtremely*) during the previous month. Recent reports suggested that a cut score of 33 could be used to determine probable PTSD (Blevins et al., 2015). The Chinese version of PCL-5 was amenable to adaptation to Chinese culture by the back-translation method (Wang et al., 2017). The measurement model of the PCL-5 was evaluated using CFA. The criteria for assessing adequate model-fit included: the NFI = 0.930, the CFI = 0.936, the IFI = 0.936, and the RFI = 0.919. The PCL-5 model was acceptable. Of note, reliability statistics for the PCL-5 in this study indicated excellent internal consistency for the PCL-5 total score in our sample (α = 0.96).

### Statistical Analysis

Data were analyzed with SPSS version 22.0. Main continuous variables were divided as categorical variables first and categorical variables were analyzed as frequency and percentage. Categorical variables were analyzed by adopting Fisher's exact test or Pearson's Chi-squared test. Univariate and multivariate logistic regression models were performed to explore potential protective factors of sociodemographic and context characteristics regarding work of preventing the COVID-19 for PTS and PD. Odds ratio (OR), adjusted OR (AOR), and 95% CI were calculated. *P*-values of <0.05 were considered statistically significant (two-sided tests).

## Results

### Sociodemographic Characteristics

The sociodemographic characteristics are shown in Table 1. Of the 1,728 samples analyzed, the females accounted for 94.4% of the total respondents. Among these samples, 1,339 (77.5%) of participants were from the frontline, most participants were in the age intervals of 20–29 (49.3%) and 30–49 years (48.4%). Most participants came from Hunan and Hubei provinces (~93.1%).

**Table 1:** Demographic characteristics of the sample (*N* = 1,728).

**Variable**	**Total (*N* = 1,728)**	**Non-PTS (*N* = 1,052)**	**PTS (*N* = 676)**	***Z/X*^**2**^**	***p*-value**	**Non-PD (*N* = 1,307)**	**PD (*N* = 421)**	***Z/X*^**2**^**	***p*-value**
**Gender**				**4.229**	**0.040**			**7.763**	**0.005**
Female	1,632 (94.4%)	984 (60.3%)	648 (39.7%)			1,223 (74.9%)	409 (25.1%)		
Male	96 (5.6%)	68(70.8%)	28(29.2%)			84(87.5%)	12 (12.5%)		
**Marital status**				**12.310**	**0.004**			3.601	0.280
Single	556 (32.2%)	367 (66.0%)	189 (34.0%)			430 (77.3%)	126 (22.7%)		
Married	1,117 (64.6%)	650 (58.2%)	467 (41.8%)			834 (74.7%)	283 (25.3%)		
Divorced	51 (3.0%)	34 (66.7%)	17 (33.3%)			41 (80.4%)	10 (19.6%)		
Widowed	4 (0.2%)	1 (25.0%)	3 (75.0%)			2 (50.0%)	2 (50.0%)		
**Age (years)**				**12.159**	**0.013**			4.872	0.281
20–29	852 (49.3%)	535 (62.8%)	317 (37.2%)			649 (76.2%)	203 (23.8%)		
30–39	597 (34.5%)	347 (58.1%)	250 (41.9%)			442 (74.0%)	155 (26.0%)		
40–49	240 (13.9%)	138 (57.5%)	102 (42.5%)			181 (75.4%)	59 (24.6%)		
50–59	35 (2.0%)	29 (82.9%)	6 (17.1%)			31 (88.6%)	4 (11.4%)		
60–69	4 (0.2%)	3 (75.0%)	1 (25.0%)			4 (100.0%)	0 (0.0%)		
**Role in pandemic prevention**				5.125	0.077			**15.282**	** <0.001**
Frontline	1339 (77.5%)	798 (59.6%)	541 (40.4%)			989 (73.9%)	350 (26.1%)		
Medical reserve corps	162 (9.4%)	101 (62.3%)	61 (37.7%)			123 (75.9%)	39 (24.1%)		
Medical routine work	227 (13.1%)	153 (67.4%)	74 (32.6%)			195 (85.9%)	32 (14.1%)		
**Exposed duration in the pandemic**				**14.331**	**0.002**			**13.297**	**0.004**
Always	241 (13.9%)	132 (54.8%)	109 (45.2%)			169 (70.1%)	72 (29.9%)		
Mostly	517 (29.9%)	299 (57.8%)	218 (42.2%)			375 (72.5%)	142 (27.5%)		
Sometimes	303 (17.5%)	179 (59.1%)	124 (40.9%)			230 (75.9%)	73 (24.1%)		
Absent	667 (38.6%)	442 (66.3%)	225 (33.7%)			533 (79.9%)	134 (20.1%)		
**Panic intensity during the COVID-19 pandemic**				**155.118**	** <0.001**			**140.771**	** <0.001**
Never	345 (20.0%)	278 (80.6%)	67 (19.4%)			307 (89.0%)	38 (11.0%)		
Occasionally	705 (40.8%)	466 (66.1%)	239 (33.9%)			570 (80.9%)	135 (19.1%)		
Sometimes	456 (26.4%)	234 (51.3%)	222 (48.7%)			319 (70.0%)	137 (30.0%)		
Often	178 (10.3%)	64 (36.0%)	114 (64.0%)			97 (54.5%)	81 (45.5%)		
Always	44 (2.5%)	10 (22.7%)	34 (77.3%)			14 (31.8%)	30 (68.2%)		
**Impact of the COVID-19 pandemic**				**152.280**	** <0.001**			**143.337**	** <0.001**
Never	253 (14.6%)	207 (81.8%)	46 (18.2%)			232 (91.7%)	21 (8.3%)		
Mild	809 (46.8%)	542 (67.0%)	267 (33.0%)			659 (81.5%)	150 (18.5%)		
Moderate	516 (29.9%)	262 (50.8%)	254 (49.2%)			347 (67.2%)	169 (32.8%)		
Severe	106 (6.1%)	29 (27.4%)	77 (72.6%)			52 (49.1%)	54 (50.9%)		
Extreme	44 (2.5%)	12 (27.3%)	32 (72.7%)			17 (38.6%)	27 (61.4%)		
**Time of browsing COVID-19-related information per day**				**19.073**	**0.001**			**17.797**	**0.001**
0–2 h	1032 (59.7%)	669 (64.8%)	363 (35.2%)			812 (78.7%)	220 (21.3%)		
3–5 h	607 (35.1%)	333 (54.9%)	274 (45.1%)			440 (72.5%)	167 (27.5%)		
6–10 h	68 (3.9%)	41 (60.3%)	27 (39.7%)			42 (61.8%)	26 38.2%)		
11–15 h	15 (0.9%)	6 (40.0%)	9 (60.0%)			9 (60.0%)	6 (40.0%)		
16–24 h	6 (0.3%)	3 (50.0%)	3 (50.0%)			4 (66.7%)	2 (33.3%)		
**Sleep**				**261.467**	** <0.001**			**296.609**	** <0.001**
Satisfactorily	925 (53.5%)	710(76.8%)	215 (23.2%)			829 (89.6%)	96 (10.4%)		
Insomnia occasionally	543 (31.4%)	264(48.6%)	279 (51.4%)			364 (67.0%)	179 (33.0%)		
Insomnia sometimes	182 (10.5%)	70(38.5%)	112 (61.5%)			102 (56.0%)	80 (44.0%)		
Insomnia frequently	66 (3.8%)	7(10.6%)	59 (89.4%)			11 (16.7%)	55 (83.3%)		
Insomnia always	12 (0.7%)	1(8.3%)	11 (91.7%)			1 (8.3%)	11 (91.7%)		
**Exercise**				**9.874**	**0.043**			**26.804**	** <0.001**
Never	732 (42.4%)	423 (57.8%)	309 (42.2%)			509 (69.5%)	223 (30.5%)		
Occasionally	606 (35.1%)	393 (64.9%)	213 (35.1%)			489 (80.7%)	117 (19.3%)		
Sometimes	228 (13.2%)	130 (57.0%)	98 (43.0%)			183 (80.3%)	45 (19.7%)		
Frequently	146 (8.4%)	95 (65.1%)	51 (34.9%)			115 (78.8%)	31 (21.2%)		
Always	16 (0.9%)	11 (68.8%)	5 (31.3%)			11 (68.8%)	5 (31.3%)		
**Diet**				**148.435**	** <0.001**			**224.467**	** <0.001**
Very poor	34 (2.0%)	5 (14.7%)	29 (85.3%)			8 (23.5%)	26 (76.5%)		
Worse	105 (6.1%)	38 (36.2%)	67 (63.8%)			38 (36.2%)	67 (63.8%)		
Average	961 (55.6%)	524 (54.5%)	437 (45.5%)			692 (72.0%)	269 (28.0%)		
Better	403 (23.3%)	299 (74.2%)	104 (25.8%)			357 (88.6%)	46 (11.4%)		
Well	225 (13.0%)	186 (82.7%)	39 (17.3%)			212 (94.2%)	13 (5.8%)		

*PTS, posttraumatic stress; PD, psychological distress. Posttraumatic stress was defined as individuals who scored 33 points in PCL-5. Psychological distress was defined as individuals who scored seven points in SRQ-20*.

*The meaning of the bold values indicates that the results are statistically significant (P-value < 0.05)*.

### Prevalence of PTS and PD Stratified by Sociodemographic Characteristics, the Influence of COVID-19, and Physical Conditions

A total of 39.12% of the participants scored above the threshold on PCL-5 (33 or more). The overall prevalence of PD (SRQ total scores > 7) was 24.36%. The prevalence of PTS and PD stratified by sociodemographic characteristics, the influence of COVID-19, and physical conditions are presented in Table 1. There was a statistically significant difference in the incidence of PTS and PD by exposed duration in the pandemic (*p* = 0.002, *p* = 0.004), the time of browsing COVID-19-related information per day (*p* = 0.001), the impact (*p* < 0.001), and panic intensity (*p* < 0.001) of COVID-19 pandemic. The incidence of PTS and PD in females was significantly higher than in males (*p* = 0.04, *p* = 0.005). The prevalence of PTS and PD was significant statistically in the diet (*p* < 0.001), exercise (*p* = 0.043, *p* < 0.001), and sleep (*p* < 0.001). There was no difference in the prevalence of PTS by the role in pandemic prevention (*p* > 0.05), and there was no statistical difference in the prevalence of PD by age (*p* > 0.05) and marital status (*p* > 0.05). Cases of PCL-5 and SRQ were more likely to have a higher level of panic, stronger subjective COVID-19 impact, frequent insomnia, and poor diet quality.

### Predictive Factors Associated With PTS and PD During the COVID-19 Outbreak

The associations of potential influence factors with PTS and PD during the COVID-19 pandemic were reported in Table 2.

**Table 2:** Logistic regression with variables predicting PTS and PD in medical staff (nurses).

	**Nurses with and without PTS**				**Nurses with and without PD**			
	**Unadjusted**		**Adjusted**		**Unadjusted**		**Adjusted**	
	**OR (95% CI)**	***p*-value**	**AOR (95% CI)**	***p*-value**	**OR (95% CI)**	***p*-value**	**AOR (95% CI)**	***p*-value**
**Marital status**		0.007		**0.002**				
Single	1 (Reference)	NA	1 (Reference)	NA				
Married	**1.395 (1.129–1.724)**	0.002	**1.582 (1.239–2.020)**	<0.001				
Divorced	0.971 (0.529–1.783)	0.924	1.137 (0.567–2.278)	0.717				
Widowed	5.825 (0.602–56.384)	0.128	6.175 (0.547–69.750)	0.141				
**Role in pandemic prevention**						0.001		**0.006**
Frontline					1 (Reference)	NA	1 (Reference)	NA
Medical reserve corps					0.896 (0.613–1.310)	0.571	1.247 (0.799–1.947)	0.330
Medical routine work					**0.464 (0.313–0.687)**	<0.001	**0.503 (0.319–0.793)**	0.003
**Panic intensity during the COVID-19 pandemic**		<0.001		** <0.001**		<0.001		**0.004**
Never	1 (Reference)	NA	1 (Reference)	NA	1 (Reference)	NA	1 (Reference)	NA
Occasionally	**2.128 (1.562–2.898)**	<0.001	**1.560 (1.097–2.217)**	0.013	**1.913 (1.301–2.814)**	0.001	1.275 (0.809–2.011)	0.295
Sometimes	**3.936 (2.848–5.442)**	<0.001	**2.175 (1.490–3.176)**	<0.001	**3.470 (2.344–5.135)**	<0.001	**1.687 (1.049–2.715)**	0.031
Often	**7.391 (4.924–11.093)**	<0.001	**3.185 (1.976–5.134)**	<0.001	**6.746 (4.311–10.558)**	<0.001	**2.489 (1.433–4.324)**	0.001
Always	**14.107 (6.638–29.981)**	<0.001	**2.648 (1.077–6.509)**	0.034	**17.312 (8.440–35.508)**	<0.001	**2.966 (1.189–7.403)**	0.020
**Impact of the COVID-19 pandemic**		<0.001		** <0.001**		<0.001		**0.013**
Never	1 (Reference)	NA	1 (Reference)	NA	1 (Reference)	NA	1 (Reference)	NA
Mild	**2.217 (1.560–3.150)**	<0.001	1.381 (0.930–2.052)	0.110	**2.515 (1.555–4.066)**	<0.001	1.431 (0.829–2.470)	0.198
Moderate	**4.363 (3.034–6.273)**	<0.001	**1.786 (1.169–2.729)**	0.007	**5.381 (3.319–8.721)**	<0.001	**1.996 (1.137–3.507)**	0.016
Severe	**11.948 (7.010–20.367)**	<0.001	**3.630 (1.963–6.711)**	<0.001	**11.473 (6.379–20.633)**	<0.001	**2.652 (1.303–5.399)**	0.007
Extreme	**12.000 (5.746–25.060)**	<0.001	**3.000 (1.262–7.130)**	0.013	**17.546 (8.259–37.275)**	<0.001	**3.115 (1.217–7.972)**	0.018
**Sleep**		<0.001		** <0.001**		<0.001		** <0.001**
Satisfactorily	1 (Reference)	NA	1 (Reference)	NA	1 (Reference)	NA	1 (Reference)	NA
Insomnia occasionally	**3.490 (2.781–4.380)**	<0.001	**2.402 (1.878–3.073)**	<0.001	**4.247 (3.219–5.601)**	<0.001	**3.033 (2.241–4.104)**	<0.001
Insomnia sometimes	**5.284 (3.778–7.389)**	<0.001	**2.786 (1.933–4.015)**	<0.001	**6.773 (4.721–9.718)**	<0.001	**3.601 (2.416–5.368)**	<0.001
Insomnia frequently	**27.834 (12.529–61.836)**	<0.001	**12.170 (5.311–27.888)**	<0.001	**43.177 (21.853–85.309)**	<0.001	**18.925 (9.156–39.114)**	<0.001
Insomnia always	**36.326 (4.663–282.963)**	0.001	**10.391 (1.169–92.391)**	0.036	**94.990 (12.131–743.790)**	<0.001	**28.725 (3.159–261.189)**	0.003
**Exercise**		0.043				<0.001		**0.047**
Never	1 (Reference)	NA			1 (Reference)	NA	1 (Reference)	NA
Occasionally	**0.742 (0.594–0.926)**	0.008			**0.546 (0.423–0.705)**	<0.001	**0.655 (0.486–0.883)**	0.005
Sometimes	1.032 (0.764–1.394)	0.837			**0.561 (0.391–0.806)**	0.002	**0.644 (0.418–0.991)**	0.045
Frequently	0.735 (0.507–1.064)	0.103			**0.615 (0.402–0.943)**	0.026	0.903 (0.545–1.494)	0.690
Always	0.622 (0.214–1.809)	0.384			1.038 (0.356–3.021)	0.946	0.678 (0.183–2.512)	0.561
**Diet**		<0.001		** <0.001**		<0.001		** <0.001**
Very poor	1 (Reference)	NA	1 (Reference)	NA	1 (Reference)	NA	1 (Reference)	NA
Worse	**0.304 (0.109–0.851)**	0.023	0.399 (0.130–1.224)	0.108	0.543 (0.224–1.317)	0.176	1.020 (0.370–2.809)	0.970
Average	**0.144 (0.055–0.375)**	<0.001	**0.242 (0.085–0.685)**	0.008	**0.120 (0.053–0.267)**	<0.001	**0.256 (0.102–0.644)**	0.004
Better	**0.060 (0.023–0.159)**	<0.001	**0.136 (0.047–0.392)**	<0.001	**0.040 (0.017–0.093)**	<0.001	**0.125 (0.047–0.332)**	<0.001
Well	**0.036 (0.013–0.099)**	<0.001	**0.112 (0.037–0.336)**	<0.001	**0.019 (0.007–0.050)**	<0.001	**0.083 (0.028–0.247)**	<0.001

*OR, odds ratio; AOR, adjusted odds ratio; PTS, posttraumatic stress; PD, psychological distress; NA, not applicable*.

*The meaning of the bold values indicates that the results are statistically significant (P-value < 0.05)*.

In the univariate logistic regression models, marital status was significantly associated with the prevalence of PTS (*p* = 0.007) in Chinese nurses, but not with PD (*p* > 0.05). The role in pandemic prevention was linked to the prevalence of PD (*p* = 0.001) in Chinese nurses, but not with PTS (*p* > 0.05). Occasional exercise was a protective factor of PTS (OR = 0.742, 95% CI = 0.594–0.926) and PD (OR = 0.546, 95% CI = 0.423–0.705) in comparison with never exercise.

In the multivariate logistic regression models, the high (often or always) panic intensity of the COVID-19 pandemic was a risk predictor of PTS (AOR = 3.185, 95% CI = 1.976–5.134) and PD (AOR = 2.489, 95% CI = 1.433–4.324) compared with low (never) panic intensity. Compared with low (*never*) impact, high (*severe or extreme*) impact of the COVID-19 pandemic was a risk predictor of PTS (AOR=3.63, 95% CI = 1.963–6.711) and PD (AOR = 2.652, 95% CI = 1.303–5.399). Contrasting to satisfactory sleep, insomnia was a risk predictor of PTS (AOR = 12.170, 95% CI = 5.311–27.888), and PD (AOR = 18.925, 95% CI = 9.156–39.114). Besides, married participants could induce an increased risk of 1.58 times to have PTS when compared with the single (AOR = 3.63, 95% CI = 1.963–6.711), but not with PD. Compared with frontline medical staff, participants engaged in daily medical work were only.5 times more likely to suffer from PD (AOR = 0.503, 95% CI = 0.319–793). The adequate (occasionally) exercise was a protective predictor of PD compared with never exercise (AOR = 0.655, 95% CI = 0.486–883), but exercise was not a predictor for PTS in the multivariate logistic regression models. High-quality diet was a protective predictor of PTS (AOR = 0.112, 95% CI = 0.037–0.336) and PD (AOR = 0.083, 95% CI = 0.028–0.247) compared with low-quality diet.

## Discussion

Our cross-sectional investigation based on the web identified the high prevalence of PTS and PD of nurses during the COVID-19 pandemic in China. In our study, the prevalence of PTS was 39.12% in nurses, higher than the Wuhan residents' prevalence of PTS (7%) a month after the COVID-19 (Liu et al., 2020). The previous study (Huang and Zhao, 2020) also showed that medical staff had a high prevalence of psychological morbidity during the outbreak compared with other professionals. Females were more susceptible to traumatic exposure, which was in line with the review of Tolin and Foa (2006). Four hundred twenty-one (24.36%) medical staff reported PD, which was in line with other reports of psychological negative changes (Huang et al., 2020). We also found that frontline were likely insidious hazards of mental health. Similarly, a study revealed that frontline HCWs had a high risk of developing psychological problems (Chen et al., 2020). Also, participants who spent too much time browsing COVID-19-related information per day were more likely to be associated with PTS and PD. Evidence of event-related potential technique indicated that heightened neural reactivity and attention toward unpleasant information, predisposed children to psychiatric symptoms when exposed to higher levels of stress, which was related to natural disasters (Kujawa et al., 2016). It was further speculated that excessive attention to negative information on the pandemic might be associated with PTS and PD.

Subsequently, this study examined protective predictors of PTS and PD. In terms of the predictors, our outcomes indicated that insomnia had been linked to more severe PTS and PD similarly (Liu et al., 2020). Except that, our study found that the married experienced higher levels of PTS than the single during the outbreak. Our results were consistent with a study in Singapore (Sim et al., 2004) which found a positive association between posttraumatic morbidities and being married. Likewise, a recent study on HCWs facing the COVID-19 pandemic showed that married, divorced, or widowed operators reported higher scores in vicarious traumatization symptoms compared with unmarried HCWs (Li Z. et al., 2020). One explanation was that married participants had more burdens of taking care of family members, following with more vulnerabilities to the COVID-19. Our study also showed that the high impact and panic intensity of the COVID-19 pandemic were risk predictors of PTS and PD. It was understandable that adequate sleep and diet improved resistance to external risk. Two of the three studies indicated that PTSD was associated with a healthier diet in female health professionals (Roberts et al., 2015; Sumner et al., 2015). Similarly, having a healthy diet was also associated with less PD in the elderly when adjusting for other lifestyle behaviors (Grønning et al., 2018). Besides, our study concluded that adequate exercise was a protective predictor of PD. There was tremendous evidence of exercise benefits (Rethorst et al., 2009; Krogh et al., 2011), it was plausible that keeping exercise improves the physical and psychological health.

Considering the present pandemic situation that COVID-19 cases are still increasing rapidly throughout the world, the quarantine in China and even in other countries would not be abolished soon. Additionally, delayed onset of traumatic symptoms might follow the stress state (Schnyder and Cloitre, 2015). Therefore, there was a concern that the prevalence of PTS among the nurses after public pandemic catastrophes would be more severe than the results of this study. Given that the survey was conducted 3 weeks following the COVID-19 pandemic, the negative changes reported likely reflected short-term and developing aspects of PTS and PD. Continuous surveillance of the psychological consequences and customized intervention for HCWs in the COVID-19 contagion should become routine as part of preparedness efforts worldwide.

This study had several limitations. Firstly, the varying gender ratios could have probably introduced gender biases into the results. Secondly, we used a web-based survey method to avoid possible infections during the outbreak of COVID-19. Future work should take account of sample gender-balancing and collection of longitudinal empirical data. Thirdly, due to our design limitations, it might be difficult to verify the veracity of the information from participants.

## Conclusion

In summary, we found that nurses suffered from significant PTS and PD during the COVID-19 pandemic. The psychological morbidity of the nurses was best understood by their physical condition, sociodemographic characteristics, and the impact and panic intensity of the COVID-19 pandemic. Low panic intensity, low level of impact, satisfactory sleep, adequate exercise, and better diet were protective factors of PTS and PD. Our results can provide directions on preventing PTS and PD in nurses. Further, it can also provide data to support clinical and psychological assistance for healthcare professionals and contribute to epidemic prevention and control work to other countries.

## Data Availability Statement

The raw data supporting the conclusions of this article will be made available by the authors, without undue reservation.

## Ethics Statement

The studies involving human participants were reviewed and approved by an ethics committee of the Second Xiangya Hospital of Central South University. The patients/participants provided their written informed consent to participate in this study. Written informed consent was obtained from the individual(s) for the publication of any potentially identifiable images or data included in this article.

## Author Contributions

DW conceived and designed the study. LX, YY, and DW performed the analysis and prepared the manuscript. All authors were involved in the study conduction and contributed substantially to its revision and approved the final manuscript.

## Conflict of Interest

The authors declare that the research was conducted in the absence of any commercial or financial relationships that could be construed as a potential conflict of interest.
