# Supplementary material for: Protective Predictors Associated With Posttraumatic Stress and Psychological Distress in Chinese Nurses During the Outbreak of COVID-19
Source: Front Psychol. 2021 May 26;12:684222. doi: 10.3389/fpsyg.2021.684222 (PMC8189291; doi:10.3389/fpsyg.2021.684222)
Supplement: Supplementary file 1 [file Data_Sheet_1.pdf]

Overview of questionnaires and items

PTSD Checklist for DSM-5 (PCL-5)

Below is a list of problems that people sometimes have in response to a very stressful experience. Please read each problem carefully and then circle one of the numbers to the right to indicate how much you have been bothered by that problem in the past month.

In the past month, how much were you bothered by:

1. Repeated, disturbing, and unwanted memories of the stressful experience?
2. Repeated, disturbing dreams of the stressful experience?
3. Suddenly feeling or acting as if the stressful experience were actually happening again (as if you were actually back there reliving it)?
4. Feeling very upset when something reminded you of the stressful experience?
5. Having strong physical reactions when something reminded you of the stressful experience (for example, heart pounding, trouble breathing, sweating)?
6. Avoiding memories, thoughts, or feelings related to the stressful experience?
7. Avoiding external reminders of the stressful experience (for example, people, places, conversations, activities, objects, or situations)?
8. Trouble remembering important parts of the stressful experience?
9. Having strong negative beliefs about yourself, other people, or the world (for example, having thoughts such as I am bad, there is something seriously wrong with me, no one can be trusted, the world is completely dangerous)?
10. Blaming yourself or someone else for the stressful experience or what happened after it?
11. Having strong negative feelings such as fear, horror, anger, guilt, or shame?
12. Loss of interest in activities that you used to enjoy?
13. Feeling distant or cut off from other people?
14. Trouble experiencing positive feelings (for example, being unable to feel happiness or have loving feelings for people close to you)?
15. Irritable behavior, angry outbursts, or acting aggressively?
16. Taking too many risks or doing things that could cause you harm?
17. Being “super alert” or watchful or on guard?
18. Feeling jumpy or easily startled?
19. Having difficulty concentrating?
20. Trouble falling or staying asleep?

The Self-Reporting Questionnaire (SRQ)

The following questions are related to certain pains and problems, that may have bothered you in the last 30 days. If you think the question applies to you and you had to describe the problem in the last 30 days, answer YES. On the other hand, if the question does not apply to you and you did not have the problem in the last 30 days, answer NO.

1. Do you often have headaches?
2. Is your appetite poor?
3. Do you sleep badly?
4. Are you easily frightened?
5. Do your hands shake?

6. Do you feel nervous, tense, or worried?
7. Is your digestion poor?
8. Do you have trouble thinking clearly?
9. Do you feel unhappy?
10. Do you cry more than usual?
11. Do you find it difficult to enjoy your daily activities?
12. Do you find it difficult to make decisions?
13. Is your daily work suffering?
14. Are you unable to play a useful part in life?
15. Have you lost interest in things?
16. Do you feel that you are a worthless person?
17. Has the thought of ending your life been on your mind?
18. Do you feel tired all the time?
19. Do you have uncomfortable feelings in your stomach?
20. Are you easily tired?

Sleep:

Since the COVID-19 outbreak, how was your sleep condition?

a. Satisfactorily
b. Insomnia occasionally
c. Insomnia sometimes
d. Insomnia frequently
e. Insomnia always

Exercise:

How have you been exercising since the COVID-19 outbreak?

a. Never
b. Occasionally
c. Sometimes
d. Frequently
e. Always

Diet:

Over the COVID-19 period, how was your diet condition?

a. Very poor
b. Worse
c. Average
d. Better
e. Well

Subjective influence of the COVID-19 pandemic:

1. How long do you expose to the COVID-19 pandemic (the time in contact with the outbreak scene)?

a. always staying in the epidemic scene
b. most of the time at the scene of the epidemic
c. a small part of the time at the scene of the epidemic
d. not at the epidemic scene

2. How long you spend browsing COVID-19-related information per day?

a.0-2 hours

b.3-5 hours
c.6-10 hours
d.11-15 hours
e.16-24 hours

3. Do you feel panic in the COVID-19 pandemic?
   a. never
   b. occasionally
   c. sometimes
   d. often
   e. always
4. To what extent has the current outbreak affected you?
   a. no impact
   b. mild impact
   c. moderate impact
   d. severe impact
   e. extreme impact

Demographic questions:

1. What is your biological sex?
   a. male
   b. female
2. How old are you?
   a.20-29
   b.30-39
   c.40-49
   d.50-59
   e.60-69
3. What is your marital status?
   a. Single
   b. Married
   c. Divorced
   d. Widowed

The role in pandemic prevention:

1. Frontline (those who directly provided services to confirmed or suspected COVID-19 patients)

2. Medical Reserve Corps (those who probably contacting confirmed or suspected COVID-19 patients)

3. Medical Routine Work (those who less likely directly servicing confirmed or suspected COVID-19 patients)
